# Antioxidant Therapies as Emerging Adjuncts in Rheumatoid Arthritis: Targeting Oxidative Stress to Enhance Treatment Outcomes

**DOI:** 10.3390/ijms26072873

**Published:** 2025-03-21

**Authors:** Rafał Bilski, Jarosław Nuszkiewicz

**Affiliations:** Department of Medical Biology and Biochemistry, Faculty of Medicine, Ludwik Rydygier Collegium Medicum in Bydgoszcz, Nicolaus Copernicus University in Toruń, 24 Karłowicza St., 85-092 Bydgoszcz, Poland

**Keywords:** antioxidant therapy, DMARDs, inflammation, molecular hydrogen, N-acetylcysteine, NF-κB, oxidative stress, reactive oxygen species, rheumatoid arthritis

## Abstract

Rheumatoid arthritis (RA) is a chronic autoimmune disorder characterized by persistent inflammation and progressive joint destruction. Recent data underscore oxidative stress as a primary factor in the pathophysiology of rheumatoid arthritis, intensifying inflammatory processes and tissue damage via the overproduction of reactive oxygen species (ROS) and compromised antioxidant defenses. Current therapies, including disease-modifying antirheumatic drugs (DMARDs), primarily target immune dysregulation but fail to address oxidative stress, necessitating novel adjunctive treatment strategies. This review explores the potential of antioxidant-based therapies as complementary approaches to RA management. Natural compounds such as curcumin, resveratrol, sulforaphane, and propolis exhibit strong anti-inflammatory and antioxidative properties by modulating redox-sensitive pathways, including nuclear factor kappa-light-chain-enhancer of activated B cells (NF-κB) and nuclear factor erythroid 2-related factor 2(Nrf2)/heme oxygenase (HO-1). N-acetylcysteine (NAC) replenishes intracellular glutathione, enhancing cellular resilience against oxidative stress. Additionally, molecular hydrogen (H_2_) selectively neutralizes harmful ROS, reducing oxidative damage and inflammation. The role of vitamin supplementation (D, B12, C, and K) in regulating immune responses and protecting joint structures is also discussed. This review aims to evaluate the efficacy and potential clinical applications of antioxidant therapies in RA, emphasizing their role in mitigating oxidative damage and improving treatment outcomes. While preliminary findings are promising, further clinical trials are needed to establish standardized dosing, long-term safety, and their integration into current RA treatment protocols.

## 1. Introduction

Rheumatoid arthritis (RA) belongs to a group of autoimmune inflammatory diseases that poses a significant health and social challenge worldwide. It affects approximately 1% of the global population, translating into millions of individuals suffering from pain, joint dysfunction, and a considerable decline in quality of life [1].

RA is more frequently diagnosed in women than in men, suggesting a potential role of hormonal factors, particularly estrogens, in its pathogenesis [2]. The hallmark symptoms include symmetric arthritis, joint swelling, and prolonged morning stiffness lasting over an hour [3]. In addition to localized joint symptoms, RA also manifests with systemic features such as chronic fatigue, weight loss, and fever, highlighting its widespread impact on overall health [4,5].

The etiology of RA is multifactorial, involving both genetic and environmental factors. Genetic predisposition is paramount, particularly the presence of specific HLA-DR4 and HLA-DR1 alleles, which elevate the likelihood of illness development [6,7].

These genes encode molecules essential for antigen presentation to the immune system, and in RA, their activity may lead to the misrecognition of self-tissues as foreign, triggering an autoimmune response [8].

Among environmental factors, smoking is one of the most significant modifiable risk factors [9]. Additionally, past bacterial and viral infections may initiate autoimmune reactions, further contributing to disease development [10,11,12]. Psychological stress and dietary patterns rich in saturated fats and processed sugars can also influence RA progression by promoting inflammatory responses and oxidative stress [13,14].

Immunological mechanisms are fundamental to understanding the onset and progression of RA. CD4+ T lymphocytes play a central role in initiating immune responses by secreting cytokines such as interleukin-17 (IL-17) and interferon gamma (IFN-γ). These cytokines promote the recruitment of inflammatory cells, including macrophages and neutrophils, which infiltrate the synovial membrane and perpetuate inflammation [15].

Under persistent inflammatory conditions, synovial fibroblasts become activated and produce matrix metalloproteinases (MMPs), enzymes responsible for the degradation of cartilage and extracellular matrix components. The uncontrolled proliferation of the synovial membrane leads to the formation of pannus, a pathological tissue that invades and destroys joint surfaces and bones, ultimately contributing to joint deformity and loss of function [16,17].

A key element in the pathogenesis of RA is the role of autoantibodies. These antibodies play a crucial role in various diseases and pathological conditions, including pregnancy loss, ankylosing spondylitis, psoriatic arthritis, and RA itself [18,19,20,21].

In RA, the most relevant autoantibodies include rheumatoid factor (RF) and anticitrullinated protein antibodies (ACPAs). These are produced in response to citrullinated proteins, which result from post-translational modifications mediated by peptidylarginine deiminase (PAD) enzymes [22]. The immune complexes formed by ACPAs further activate the complement system, intensifying tissue damage and inflammation. This process creates a self-perpetuating cycle, where each step amplifies the inflammatory response, leading to progressive joint destruction [23,24].

In recent years, increasing attention has been given to the role of oxidative stress in RA pathogenesis. Oxidative stress results from an imbalance between the production of reactive oxygen species (ROS) and the body’s ability to counteract their harmful effects using antioxidant systems. ROS, such as hydrogen peroxide (H_2_O_2_), the superoxide anion radical (O_2_•^−^), and the hydroxyl radical (•OH), are normal byproducts of metabolic processes. While they serve essential functions in cell signaling and immune defense, their excess leads to damage to proteins, lipids, and DNA [25].

In RA, oxidative stress not only fuels inflammatory pathways but also contributes to joint tissue degradation, promoting disease progression and exacerbating clinical symptoms [26,27]. A significant source of ROS production is activated immune cells, including neutrophils and macrophages, which release large amounts of ROS in response to proinflammatory cytokines, such as TNF-α and IL-6. Elevated ROS levels, in turn, trigger redox-sensitive signaling pathways, particularly NF-κB and MAPK, which further enhance the transcription of proinflammatory cytokines and chemokines.

This interplay between oxidative stress and inflammation creates a vicious cycle, where inflammation drives ROS overproduction, and oxidative stress further amplifies inflammatory damage [28,29]. Within the RA context, ROS also play a pivotal role in cartilage and bone destruction. Increased ROS activity leads to the activation of metalloproteinases (MMPs) and promotes osteoclast differentiation, both of which contribute to bone resorption and the progressive loss of joint function [30,31,32,33].

In summary, RA is a multifaceted disease in which autoimmune, inflammatory, and oxidative processes are deeply interconnected, leading to sustained tissue damage. A thorough understanding of these mechanisms, particularly the role of oxidative stress, is essential for the development of new, more effective therapeutic strategies aimed at both controlling inflammation and protecting joint structures.

This review aims to provide a comprehensive evaluation of the efficacy and potential clinical applications of antioxidant therapies in RA, emphasizing their role in mitigating oxidative damage, modulating key inflammatory pathways, and enhancing treatment outcomes. Unlike previous reviews, this paper integrates recent advancements in understanding the Nrf2/Keap1 (Kelch-like ECH-associated protein 1—nuclear factor (erythroid-derived 2)-like 2) axis, AGE-RAGE (advanced glycation end product–advanced glycation end-product receptor) signaling, and oxidative stress-induced immune dysregulation, highlighting their implications in RA pathophysiology. Additionally, it presents an updated analysis of emerging antioxidant interventions, including sulforaphane, resveratrol, molecular hydrogen, and targeted vitamin therapies, critically assessing their therapeutic potential, limitations, and translational applicability in clinical settings. By addressing these aspects, this review contributes to the identification of novel therapeutic targets and provides insights into the future direction of antioxidant-based interventions in RA management.

## 2. Changes in Oxidative Stress Parameters in RA

Scientific studies have clearly confirmed that oxidative stress plays a central role in the pathogenesis of RA. Significant changes in the levels of oxidative stress markers and antioxidant enzyme activity have been demonstrated in patients with RA compared to healthy individuals. One of the most important indicators of oxidative stress is malondialdehyde (MDA), a product of lipid peroxidation [34]. In patients with RA, elevated serum MDA levels were found, which indicates increased damage to cell membranes by reactive oxygen species. High MDA levels correlate with the severity of clinical symptoms, such as pain and morning stiffness, which emphasizes its importance as a biomarker of disease activity [35,36]. Another key parameter is 8-hydroxydeoxyguanosine (8-OHdG), a marker of DNA damage induced by ROS [37]. Studies have shown that 8-OHdG levels are significantly elevated in the urine and serum of RA patients, suggesting that oxidative stress affects genome integrity in synovial cells and other tissues involved in the disease process. Increased 8-OHdG levels may also be associated with increased inflammatory processes and progressive joint destruction [38,39]. Redox imbalance in RA is also evident in the reduced activity of antioxidant enzymes such as superoxide dismutase (SOD), catalase (CAT), and glutathione peroxidase (GPx) [39]. These enzymes play a key role in neutralizing ROS, but their reduced activity in RA patients leads to the accumulation of reactive oxygen species and increased oxidative stress. For example, the activity of SOD, responsible for the conversion of the superoxide anion radical to the less harmful hydrogen peroxide, is significantly reduced in patients with RA, which increases susceptibility to oxidative damage [40,41].

Other studies indicate reduced availability of glutathione (GSH), one of the most important intracellular antioxidants. Glutathione is involved in the neutralization of H_2_O_2_ and other reactive oxygen species, and its deficiency leads to increased lipid peroxidation and protein damage. Reduced glutathione levels in the synovial tissues and serum of patients with RA are associated with disease progression and increased inflammatory symptoms [42,43,44].

**Figure 1:**
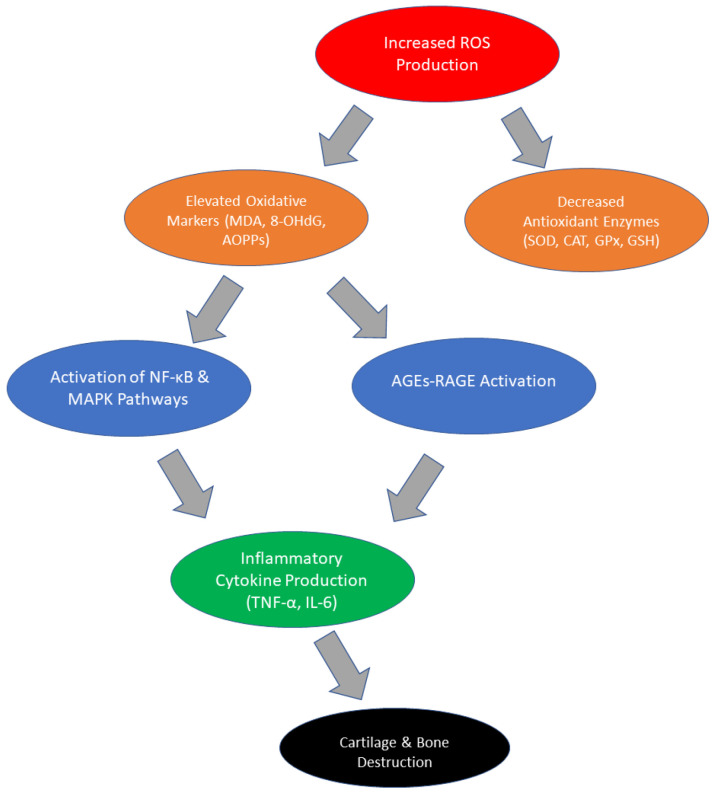
Pathophysiological changes in RA patients due to oxidative stress.

Another interesting aspect is the role of advanced oxidation protein products (AOPPs), which are formed as a result of the reaction of proteins with ROS. AOPPs are not only markers of oxidative stress but also active mediators of inflammatory processes. Their levels are significantly elevated in patients with RA and correlate with the levels of proinflammatory cytokines such as IL-6 or TNF-α. AOPPs can stimulate cytokine production by neutrophils, creating a positive feedback loop that increases tissue damage [45,46]. AGEs and their receptors (RAGEs) play a particularly important role in the context of RA. AGEs are a group of compounds formed due to the non-enzymatic reaction of glucose with proteins, lipids, or nucleic acids, which leads to a change in their biological functions [46,47]. High concentrations of AGEs are observed in patients with RA, and their accumulation contributes to oxidative and inflammatory damage. AGEs bind to RAGEs, which are present on the surface of various cell types, including macrophages and synovial fibroblasts. Activation of RAGEs by AGEs leads to increased ROS production, which drives inflammatory processes and increases the destruction of joint tissues [48,49,50]. Increased RAGE expression in the synovium of patients with RA correlates with the severity of clinical symptoms, such as pain and morning stiffness. Moreover, the activation of the AGE-RAGE pathway is associated with the activation of NF-κB, a key transcriptional regulator of proinflammatory genes [48]. This pathway enhances the production of cytokines such as TNF-α and IL-6, which further contributes to disease progression. Pharmacological interventions aimed at blocking the AGE-RAGE pathway, e.g., using RAGE inhibitors, are currently being investigated as potential therapeutic strategies in RA [51,52,53]. In summary, changes in oxidative stress parameters in RA include both increased levels of oxidative damage markers such as MDA, 8-OHdG, and AOPPs, as well as the activation of the AGE-RAGE pathway, which drives inflammatory processes, and the decreased activity of antioxidant enzymes such as SOD, CAT, and GPx. Figure 1 presents a summary of pathophysiological changes in RA patients due to oxidative stress [Figure 1]. These disorders not only reflect the intensification of inflammatory processes but also contribute to the progression of the disease by amplifying the mechanisms of tissue destruction. A summary of changes in oxidative stress parameters in RA is provided in Table 1.

## 3. Current Therapeutic Options and Their Limitations

Current therapies for RA include a wide range of drugs, including DMARDs, nonsteroidal anti-inflammatory drugs (NSAIDs), and glucocorticosteroids. These drugs, although effective, have significant limitations that may affect their long-term effectiveness and tolerability in patients [67,68].

DMARDs are divided into conventional synthetic (csDMARDs), biologic (bDMARDs), and targeted synthetic (tsDMARDs) DMARDs [68]. Among csDMARDs, methotrexate (MTX) remains the gold standard due to its effectiveness in controlling inflammation and preventing disease progression. Other csDMARDs, such as leflunomide, sulfasalazine, and hydroxychloroquine, are commonly used either alone or in combination therapy. However, despite their widespread use, csDMARDs have notable limitations, including a delayed onset of action, gastrointestinal intolerance, hepatotoxicity, and myelosuppression. Moreover, 20–30% of patients fail to achieve adequate disease control with csDMARDs alone, necessitating escalation to bDMARDs [68,69,70,71].

bDMARDs, including TNF inhibitors (etanercept, infliximab, adalimumab), IL-6 inhibitors (tocilizumab), B-cell-depleting agents (rituximab), and T-cell co-stimulation blockers (abatacept), have significantly advanced RA management [72]. These therapies target specific proinflammatory pathways, offering greater efficacy in MTX-resistant patients. Despite their benefits, bDMARDs present significant challenges, including high treatment costs, parenteral administration, and an increased risk of infections, particularly tuberculosis and opportunistic infections [73,74,75]. The immunosuppressive effects of bDMARDs raise concerns about long-term safety, necessitating careful patient monitoring. Additionally, some patients develop resistance to bDMARDs due to the formation of neutralizing antibodies, which further complicates treatment strategies [73].

A more recent class of RA therapies, tsDMARDs, such as Janus kinase (JAK) inhibitors (tofacitinib, baricitinib, upadacitinib), allows for oral administration and has a rapid onset of action compared to bDMARDs. These agents inhibit intracellular cytokine signaling, offering an alternative for patients with an inadequate response to csDMARDs or bDMARDs. However, concerns have emerged regarding thromboembolic events, cardiovascular risks, and malignancy, prompting regulatory agencies to introduce cautionary guidelines on their long-term use [76,77].

### Nonsteroidal Anti-Inflammatory Drugs (NSAIDs) and Glucocorticosteroids

NSAIDs, such as ibuprofen and celecoxib, are used to reduce pain and inflammation by inhibiting cyclooxygenase (COX) activity. Although they provide symptomatic relief, they do not affect the course of the disease or prevent joint destruction. Long-term use of NSAIDs is associated with a risk of gastrointestinal toxicity, nephrotoxicity, and cardiovascular complications, especially in patients with comorbidities [78,79,80]. Glucocorticoids, such as prednisone, are potent anti-inflammatory and immunosuppressive agents. Their use in the short term can provide significant symptomatic relief, but long-term therapy leads to serious side effects, such as osteoporosis, hypertension, diabetes, and an increased risk of infection. Furthermore, discontinuation of glucocorticoids after long-term use is difficult because of the risk of adrenal insufficiency [81,82,83].

One of the major challenges in developing effective adjuvant therapies for RA is the difficulty of delivering active compounds to the inflamed joint tissues due to immunological and pharmacokinetic barriers. Conventional pharmacotherapies, including synthetic and biological DMARDs, often exhibit limitations related to poor bioavailability, systemic toxicity, and off-target effects, which can compromise their long-term efficacy and safety. Recent advances in nanomedicine offer promising strategies to overcome these challenges by employing nanoscale drug delivery systems that enhance the solubility, stability, and bioavailability of therapeutic agents while enabling targeted delivery to inflamed synovial tissues. Nanocarriers, such as liposomes, polymeric nanoparticles, and solid lipid nanoparticles, can facilitate the passive or active accumulation of drugs in inflamed joints, minimizing systemic exposure and adverse effects [84].

Present therapeutic alternatives for RA encompass NSAIDs, glucocorticoids, and DMARDs. In the last twenty years, the emergence of biological DMARDs and tailored synthetic DMARDs has markedly enhanced the management of rheumatoid arthritis. Nonetheless, a significant proportion of RA patients continue to be unable to achieve and maintain clinical remission, highlighting the pressing necessity for novel therapeutic approaches. Another problem is the high variability in treatment response, which often requires a “trial-and-error” approach in selecting therapy. The costs of treatment, especially biological drugs, are an additional burden for patients and healthcare systems. Another challenge is the lack of treatments targeting the mechanisms of oxidative stress, which plays a key role in the pathogenesis of RA. Current drugs mainly focus on immune modulation and a reduction in inflammation, omitting redox balance. As a result, they do not effectively reduce oxidative damage, which can lead to further joint destruction despite controlling inflammation. Furthermore, nanotheranostic platforms integrating both diagnostic and therapeutic functionalities could revolutionize RA management by allowing real-time monitoring of disease progression and treatment response. The incorporation of nanotechnology into RA therapy presents a significant opportunity to improve clinical outcomes and address the unmet needs of current pharmacological approaches, particularly by enhancing drug retention at the disease site and reducing immune-mediated clearance.

Therefore, it is necessary to develop therapeutic strategies that suppress inflammation and protect tissues from oxidative damage.

## 4. Potential Antioxidant Therapies for RA

Antioxidants are one of the most promising groups of compounds in the adjunctive therapy of RA because oxidative stress plays a central role in the pathogenesis of this disease. Antioxidant therapies aim not only to neutralize ROS but also to modulate key signaling pathways that increase inflammation and tissue destruction. Their use as an adjunct to standard therapies, such as DMARDs, may be beneficial by improving treatment outcomes and reducing adverse effects [85,86].

### 4.1. Curcumin

Curcumin, the main bioactive component of turmeric (*Curcuma longa*), has potent anti-inflammatory and antioxidant properties, making it a potential candidate for adjunctive therapy in RA. Its therapeutic action is based on several key mechanisms, including inhibition of the NF-κB pathway, activation of the Nrf2/HO-1 pathway, and regulation of proinflammatory cytokines [87,88]. One of the most important mechanisms of action of curcumin is the inhibition of the transcription factor NF-κB, which plays a key role in regulating the inflammatory response. NF-κB is activated under conditions of chronic inflammation, leading to excessive production of proinflammatory cytokines such as TNF-α, IL-6, and IL-1β. The Nrf2/Keap1 signaling pathway is a crucial regulator of cellular redox homeostasis, playing a protective role against oxidative damage by modulating the expression of antioxidant and cytoprotective genes. Dysregulation of this pathway has been implicated not only in RA but also in various pathological conditions, including cancer and neurodegenerative diseases, highlighting its broad impact on inflammation and oxidative stress-driven disorders [89,90]. Curcumin blocks the phosphorylation and degradation of IκBα—an NF-κB inhibitor—which prevents the translocation of the p65 subunit to the cell nucleus and reduces the expression of genes responsible for maintaining the inflammatory state in RA [91,92]. Curcumin also acts as a potent antioxidant by activating the Nrf2/HO-1 pathway. Nrf2 is a key transcription factor regulating the expression of antioxidant enzymes such as SOD, CAT, and GPx. Under conditions of oxidative stress, curcumin promotes the dissociation of Nrf2 from the inhibitory protein Keap1, allowing its translocation to the nucleus and increased transcription of protective enzymes. By this mechanism, curcumin reduces ROS levels and protects cells from oxidative damage, which is an essential pathogenic factor in RA [88,93]. Another important mechanism of action of curcumin is its effect on cytokine balance. Curcumin reduces the level of IL-17, a cytokine produced by Th17 lymphocytes, which contributes to the intensification of the autoimmune process and joint destruction. At the same time, it increases the activity of regulatory T lymphocytes (Treg), which may help control the overactive immune response in RA [94]. Meta-analyses of clinical studies have shown that curcumin supplementation leads to a reduction in inflammatory markers, such as C-reactive protein (CRP) and the erythrocyte sedimentation rate (ESR), and improves clinical outcomes, including a decrease in the number of swollen and tender joints and an improvement in the DAS28 disease activity index. Due to its anti-inflammatory and antioxidant properties, curcumin may be a valuable addition to standard RA therapy, especially in the context of minimizing the side effects of conventional antirheumatic drugs (DMARDs). However, due to the low bioavailability of curcumin, its effectiveness may be increased by using special formulations, such as nanoparticles, phospholipid phytosomal complexes, or combinations with piperine, which increases its absorption [95].

### 4.2. Resveratrol and Other Polyphenols

Resveratrol, a polyphenol found in grapes and red wine, is another compound with therapeutic potential in RA. Its action is based on reducing osteoclast activation, which prevents bone resorption, and inhibiting the NF-κB and MAPK pathways, which leads to a reduction in the production of proinflammatory cytokines. Resveratrol also activates the phosphatidylinositol 3-kinase (PI3K)/protein kinase B (AKT) pathway, which plays a key role in regulating the inflammatory response. Studies have shown that PI3K/Akt stimulation leads to the phosphorylation and inactivation of the transcription factor forkhead box protein O1 (FoxO1). FoxO1, in its active form, increases the expression of TLR4, which increases inflammation. Inactivation of FoxO1 by resveratrol causes a decrease in TLR4 levels, which results in a weakened inflammatory response [96,97]. Resveratrol also supports mitochondrial activity and reduces ROS accumulation in synovial fibroblasts. In RA, there is excessive production of MMPs, which degrade joint cartilage and lead to bone destruction. In addition, the activation of silent information regulator sirtuin 1 (SIRT1) by resveratrol inhibits the expression of metalloproteinases 1 and 13, which limits extracellular matrix degradation and disease progression. Clinical studies suggest that resveratrol supplementation may reduce the severity of RA symptoms, but long-term safety and efficacy require further studies [98,99].

In addition to resveratrol, other polyphenols have shown promise as potential adjunctive therapies in RA due to their antioxidant and anti-inflammatory properties. Quercetin, a flavonoid found in onions, apples, and berries, has been reported to inhibit NF-κB signaling and reduce the production of proinflammatory cytokines, including TNF-α and IL-6. In vitro and in vivo studies suggest that quercetin can attenuate oxidative stress by enhancing the activity of antioxidant enzymes such as SOD and CAT, which are often dysregulated in RA [100]

Another promising polyphenol is epigallocatechin gallate (EGCG), the primary catechin found in green tea. EGCG exhibits potent immunomodulatory effects, particularly by inhibiting fibroblast-like synoviocyte (FLS) activation and suppressing the expression of MMPs, which contribute to cartilage degradation in RA. Additionally, EGCG has been shown to promote apoptosis in synovial cells, reducing hyperplasia of the synovial lining, a hallmark of RA pathology [101].

Pomegranate extract, rich in ellagitannins, has also demonstrated protective effects in RA by reducing oxidative stress and inflammation. Studies have indicated that pomegranate polyphenols suppress TNF-α and IL-1β production while enhancing HO-1 expression via the Nrf2 pathway. This mechanism aligns with the therapeutic potential of other polyphenols in reinforcing endogenous antioxidant defenses [100].

These additional polyphenols complement resveratrol’s effects by targeting similar oxidative and inflammatory pathways while offering alternative mechanisms of immune modulation. Their potential inclusion in RA treatment strategies warrants further clinical investigation to optimize their efficacy as adjunctive therapies alongside conventional DMARDs.

### 4.3. N-Acetylcysteine (NAC)

NAC has a potential therapeutic effect on RA due to its antioxidant and anti-inflammatory properties. As a precursor of glutathione, one of the most important intracellular antioxidants, NAC helps to increase its level, which allows for more effective neutralization of reactive oxygen species such as hydroxyl radicals, hypochlorous acid, and hydrogen peroxide [102,103,104]. Additionally, NAC can inhibit the activation of the transcription factor NF-κB, which plays a key role in regulating inflammation. Thanks to this, NAC limits the production of inflammatory mediators, which reduces the overall inflammation in the joints. In addition, in vivo studies proved that the use of NAC correlates with a decrease in the level of CRP and ESR, which are clinical markers of inflammatory activity in RA [105,106]. Their reduction may contribute to improving disease symptoms, such as joint pain and swelling, which indicates the potential benefits of NAC supplementation in adjunctive therapy. Another important mechanism of action of NAC is its influence on the function of synovial fibroblasts, which, in RA, exhibit an aggressive phenotype and contribute to joint destruction through excessive production of MMPs. NAC inhibits their activity, which limits the degradation of the extracellular matrix and the destruction of joint cartilage, reducing disease progression in animal models [107]. In addition to animal models, clinical trials proved that NAC affects the balance between osteoblasts and osteoclasts, which may be important in protecting bone tissue from resorption. Overactive osteoclasts in RA lead to bone damage, and NAC may limit this process by regulating the signals responsible for their differentiation and resorption function [108,109].

### 4.4. Sulforaphane (SFN)

SFN, a natural isothiocyanate present in cruciferous vegetables, has strong anti-inflammatory and antioxidant properties, making it a potential candidate for supportive therapy in RA. Its action is mainly due to the activation of the Nrf2 transcription pathway, inhibition of the NF-κB pathway, and regulation of the immune response through its effect on B and T lymphocytes. The activation of the Nrf2 transcription factor by sulforaphane leads to the increased expression of antioxidant enzymes such as heme oxygenase 1 (HO-1) and NAD(P)H:quinone oxidoreductase 1 (NQO1) [110,111,112,113,114]. These enzymes neutralize reactive oxygen species (ROS) and protect cells from oxidative stress, which, in RA, plays a key role in the exacerbation of inflammation and tissue damage. Sulforaphane induces oxidative stress in endothelial cells by activating the Nrf2 pathway, which upregulates antioxidant defense mechanisms while simultaneously modulating mitochondrial bioenergetics. This dual effect can lead to increased proton leakage and alterations in mitochondrial respiration, potentially contributing to redox imbalance and endothelial dysfunction under specific conditions [115]. In addition, an animal model proved that SFN inhibits the NF-κB pathway, which is the main regulator of inflammation and is responsible for the production of proinflammatory cytokines such as TNF-α, IL-6, and IL-17. Studies have shown that SFN reduces the levels of these cytokines, which translates into a reduction in the intensity of the inflammatory process in the joints. In addition, sulforaphane affects B lymphocytes and their differentiation, which is important in the context of RA pathogenesis, where the excessive activation of these cells leads to the production of autoantibodies, such as RF and anti-CCP. In animal models, SFN has been shown to inhibit the differentiation of B lymphocytes into plasma cells and reduce the production of autoantibodies, which may limit the autoimmune components of RA [114]. According to in vitro studies, SFN affects the Th17 lymphocyte population by inhibiting the transcription factor RORγt, which reduces the production of IL-17—one of the key cytokines driving the inflammatory process in RA. An interesting mechanism of SFN action is its ability to increase the level of ROS in T cells, which, in controlled conditions, leads to their suppression and limits their ability to induce inflammation. At the same time, SFN reduces the level of GSH, which leads to changes in the redox balance and the inhibition of proinflammatory functions of T lymphocytes [116]. In summary, sulforaphane may be a promising therapeutic option in RA, acting on multiple levels—from reducing oxidative stress through regulating immune cell function to inhibiting key inflammatory pathways. Its multidirectional action makes it a valuable addition to standard therapies, such as DMARDs, especially in the context of reducing side effects and improving therapeutic response in RA patients.

### 4.5. Propolis

Propolis, a natural substance produced by bees, has potent anti-inflammatory and antioxidant properties, making it a potential candidate for supportive therapy in RA. One of the key mechanisms of action of propolis is the inhibition of NF-κB activation, a major regulator of the inflammatory process. NF-κB plays an essential role in the synthesis of proinflammatory cytokines, such as TNF-α, IL-6, and IL-17, which drive the autoimmune process and joint tissue destruction. Propolis, by inhibiting the transcription of these mediators, reduces inflammation and improves the functioning of synovial cells [117,118]. Another critical aspect of propolis action is its effect on oxidative stress, which, in RA, contributes to cartilage damage and joint tissue degradation. Propolis supplementation has been shown to increase the activity of antioxidant enzymes, such as SOD, CAT, and GPx, while reducing the levels of oxidative stress markers, such as MDA. Thanks to this, propolis neutralizes ROS, which reduces cellular damage and limits disease progression [119,120,121]. In addition, propolis modulates the immune response by influencing the activity of T and B lymphocytes, which play a key role in the pathogenesis of RA. Studies in both animal models and clinical trials have shown that propolis can inhibit the activity of Th17 lymphocytes, which are responsible for the production of IL-17—a key cytokine driving the inflammatory process and cartilage destruction. At the same time, propolis supports Treg, which may lead to better control of the overactive immune response [122,123,124]. In clinical trials, propolis supplementation in patients with RA has shown potential benefits in the form of lower levels of inflammatory markers, such as CRP, and reduced the severity of disease symptoms. The clinical trials demonstrated that stingless bee propolis neither enhanced the quality of life in rheumatoid arthritis patients nor diminished disease activity. The ineffectiveness of propolis in mitigating disease activity was linked to the drugs administered to the individuals before the clinical investigation. Conversely, Brazilian propolis has been documented to exert beneficial effects in reducing the activity of rheumatoid arthritis in mice, indicating that propolis may offer innovative therapeutic alternatives [125,126]. Although these mechanisms require further clinical investigation, these results suggest that propolis may be a valuable adjunct to RA therapy.

### 4.6. Molecular Hydrogen Therapies

Molecular hydrogen (H_2_) is a unique antioxidant with broad therapeutic potential. Its antioxidant activity is based on the selective neutralization of ROS, especially the most toxic hydroxyl radicals (•OH) and peroxynitrite (ONOO^−^), while not affecting other, less reactive and physiologically relevant ROS, such as hydrogen peroxide (H_2_O_2_) or nitric oxide (NO•) [127,128]. This selectivity allows hydrogen to protect cells from oxidative stress without interfering with important signaling pathways that require the presence of some ROS. Its antioxidant activity is unique due to its ability to rapidly diffuse across cell membranes, allowing it to penetrate cells and reach mitochondria and cell nuclei. As a result, hydrogen can protect both DNA from oxidative damage and mitochondria from dysfunctions resulting from excessive ROS production. One of the key mechanisms of action of H₂ is its ability to modulate the expression of genes involved in antioxidant defense and inflammation reduction. It stimulates the Nrf2 pathway, which controls the expression of antioxidant enzymes such as CAT, SOD, and GPx. In this way, H_2_ enhances the body’s natural defense mechanisms against oxidative stress, which was proven by clinical trials. [129,130]. In addition, hydrogen has anti-inflammatory effects by inhibiting the activation of the NF-κB pathway, which is a key regulator of the inflammatory response. NF-κB is responsible for the expression of proinflammatory cytokines, such as TNF-α, IL-6, and IL-1β, which intensify pathological processes in chronic diseases, including RA. H_2_ limits their production, reducing inflammation and slowing down disease progression. An interesting aspect of hydrogen therapy is the variety of methods of its administration, including hydrogen inhalation, drinking hydrogen-saturated water, injections of hydrogen-enriched saline, or the use of hydrogen-rich water baths. This allows for tailoring the therapy to the individual needs of patients and optimizing the bioavailability of this gas [131,132,133,134]. Molecular hydrogen has potent antioxidant and anti-inflammatory effects by neutralizing harmful ROS, activating the Nrf2 pathway, and inhibiting NF-κB. Its ability to diffuse rapidly and its lack of side effects make it a promising candidate for adjuvant therapy in conditions associated with oxidative stress, including RA.

### 4.7. The Role of Vitamins in Antioxidant Therapy

Vitamins play an important role in modulating inflammatory processes and protecting joint structures in RA. Clinical trials have shown that, in particular, vitamins D, B12, C, and K exhibit properties that can support standard therapies and alleviate disease symptoms through immunomodulatory, antioxidant, and bone and cartilage protective effects [135].

Vitamin D (VD) plays a key role in regulating the immune system and reducing inflammation in RA. Its deficiency is common in patients with this disease and is associated with greater severity of symptoms and higher levels of inflammatory markers. The active form of VD (calcitriol) inhibits the production of proinflammatory cytokines, such as TNF-α, IL-6, and IL-17, while supporting the function of Tregs, which help control the body’s autoimmune response. In addition, vitamin D has a protective effect on bone tissue, preventing osteoporosis and bone demineralization, which often accompany RA. Moreover, its supplementation may improve the efficacy of methotrexate (MTX) therapy by reducing the adverse side effects of this drug, such as liver toxicity and bone loss [136,137,138,139,140].

Vitamin B12 (B12) is important in metabolic and neurological processes, and its deficiency is common in patients with RA, especially those treated with MTX. The deficiency of this vitamin leads to increased homocysteine levels, which can increase inflammation and damage to the vascular endothelium. In addition, low B12 levels can worsen symptoms of fatigue, which are a common problem in RA [141,142]. Supplementation with B12, especially in the form of methylcobalamin, may not only improve the hematological status of patients but also affect nerve regeneration and reduce pain [143,144].

Vitamin C (VC) is a powerful antioxidant that neutralizes the ROS responsible for the oxidative stress that increases inflammation in RA. As a cofactor in collagen synthesis, it plays a key role in maintaining the integrity of connective tissue, which may help protect articular cartilage from degradation [145]. Additionally, vitamin C has immunomodulatory effects, affecting the activity of T lymphocytes and macrophages. Studies suggest that high doses of vitamin C can reduce levels of inflammatory markers such as CRP, which may translate into reduced disease symptoms. However, excess vitamin C can lead to side effects such as kidney stones, so precise dosing is necessary [146,147,148]. Vitamin K (VK) plays an important role in maintaining bone health and regulating inflammatory processes. It is essential for the activation of proteins such as osteocalcin and Matrix Gla-Protein (MGP), which are involved in bone mineralization and the inhibition of pathological calcification of soft tissues, including joints. VK deficiency is associated with an increased risk of osteoporosis and fractures, which is particularly important for patients with RA, who experience accelerated bone loss. In addition, vitamin K affects the immune system by inhibiting osteoclast activity and reducing the level of inflammatory mediators, which may be beneficial in controlling inflammation in RA [149,150,151,152]. Vitamins D, B12, C, and K play important roles in RA therapy by reducing inflammation, protecting bone and cartilage, and supporting the immune system. Their supplementation may be particularly beneficial as an adjunct to standard treatment, improving the effectiveness of therapy and mitigating its side effects. However, further studies are needed to determine the optimal doses and supplementation regimens for RA patients.

## 5. The Importance of Antioxidant Therapies

Antioxidant therapies have the potential to become an important complement to standard RA treatment. By neutralizing ROS, modulating signaling pathways, and protecting tissues from oxidative damage, they can help reduce the severity of symptoms, improve the efficacy of therapy, and reduce the side effects of conventional drugs. Combining antioxidants with existing therapies opens up new possibilities in the treatment of RA and emphasizes the need for further research in this area. A summary of potential antioxidant therapies is presented in Table 2.

Oxidative stress plays a key role in the pathogenesis of RA, intensifying inflammatory processes and joint tissue destruction. The antioxidant therapies presented in this paper show great potential in the context of supporting the treatment of this disease. Their ability to neutralize ROS and modulate signaling pathways is crucial, which results in reduced inflammation and limited tissue destruction. The literature review indicates that natural compounds such as curcumin, resveratrol, NAC, sulforaphane, and propolis can effectively reduce oxidative stress and modulate the immune response. Their use in clinical practice could improve the efficacy of standard therapies while reducing the risk of adverse events. Of particular interest are therapies combining antioxidants with DMARDs, which can enhance the therapeutic effect and improve treatment tolerance.

Therapies using molecular hydrogen and supplementation with vitamins such as C, E, and D are other promising approaches that require further clinical trials. Despite promising results, challenges such as the limited bioavailability of natural antioxidants and the lack of standardization of their use still pose significant barriers to the full implementation of these therapies in clinical practice. Another problem is the diverse responses of patients to different antioxidant therapies, which may result from individual differences in metabolism, genetics, or diet. Further clinical trials are needed to better understand which patient groups may benefit the most from these therapies. The long-term effects of antioxidant therapies in RA are still unclear. In particular, it remains to be assessed whether their use in combination with DMARDs, NSAIDs, and glucocorticosteroids can lead to synergistic benefits without increasing the risk of adverse effects. Furthermore, the development of modern technologies such as nanoparticles or targeted drug carriers can increase the bioavailability and efficacy of antioxidants, making these therapies even more promising.

## 6. Conclusions and Future Directions

Oxidative stress is a key element in the pathogenesis of RA, contributing to the exacerbation of inflammatory processes and the destruction of joint tissues. Antioxidant therapies, both pharmacological and dietary, are promising approaches to supporting standard RA treatment. Natural compounds such as curcumin, resveratrol, NAC, sulforaphane, and propolis show the potential to reduce oxidative stress and modulate the immune response. In parallel, vitamin supplementation and molecular hydrogen therapies can support redox balance and improve the overall health of patients. Although the results of previous studies are promising, further clinical studies are needed to better understand the mechanisms of antioxidant action and determine their place in therapeutic strategies for RA. In the future, the development of antioxidant therapies in RA may lead to significant progress in the treatment of this disease, especially in the context of personalized medicine, where therapies are tailored to the individual needs of the patient. The ultimate goal is not only to control disease symptoms but also to improve quality of life and prevent long-term complications associated with RA.

## Figures and Tables

**Table 1:** Changes in antioxidants and oxidative stress markers levels in rheumatoid arthritis (RA) patients.

Parameter	Levels in RA Patients	References
Total Oxidant Status (TOS)	Elevated	[54,55,56]
Total Antioxidant Status (TAS)	Decreased	[54,55,56]
Oxidative Stress Index (OSI)	Elevated	[54,55,56]
MDA	Elevated	[57,58,59,60,61,62,63]
8-OHdG	Elevated	[37,38,39]
SOD	Decreased	[37,41,59,63]
	Elevated	[57,58,61]
GSH	Decreased	[42,43,44,61,62,64]
	Elevated	[57,63]
CAT	Decreased	[59,65]
GPx	Decreased	[62,63,65]
	Elevated	[57,61,66]
AOPPs	Elevated	[45,46]
AGEs	Elevated	[46,47,48,49,50]
RAGE	Elevated	[48,51,52,53]

**Table 2:** Summary of potential antioxidant therapies in rheumatoid arthritis (RA).

Substance	Mechanism of Action	Potential Benefits in RA	References
Curcumin	Inhibition of NF-κB, activation of Nrf2/HO-1, regulation of proinflammatory cytokines (TNF-α, IL-6, IL-1β)	Reduction in inflammation, alleviation of clinical symptoms, enhancement of DMARD efficacy	[88,91,92]
Resveratrol	Inhibition of osteoclast activation, suppression of NF-κB and MAPK, activation of PI3K/Akt, inhibition of TLR4 expression	Protection against bone resorption, reduction in extracellular matrix degradation, attenuation of inflammatory response	[96,97,98,99]
NAC	Precursor of glutathione, ROS neutralization, NF-κB inhibition, regulation of matrix metalloproteinases (MMPs)	Improvement in redox balance, reduction in oxidative stress, slowdown of joint destruction	[102,103,104,105]
Sulforaphane	Activation of Nrf2, inhibition of NF-κB, regulation of B and T lymphocytes, reduction in IL-17	Decrease in autoantibody production, synovial membrane protection, reduction in IL-17	[110,111,112,113]
Propolis	Inhibition of NF-κB, enhancement of antioxidant enzyme activity (SOD, CAT, GPx), modulation of Th17 and Treg lymphocytes	Reduction in oxidative damage, lowering of inflammatory markers, improvement of joint function	[117,118,119]
Molecular hydrogen	Neutralization of hydroxyl radicals (•OH) and peroxynitrite (ONOO^−^), activation of Nrf2, inhibition of NF-κB	Protection against DNA and mitochondrial damage, reduction in inflammatory markers, enhancement of cellular immunity	[127,128,129,131]
Vitamin D	Regulation of proinflammatory cytokines (TNF-α, IL-6, IL-17), bone protection, support for methotrexate therapy	Reduced osteoporosis risk, enhanced immune response, decreased methotrexate side effects	[136,137,138,139]
Vitamin B12	Reduction in homocysteine levels, nerve regeneration, fatigue reduction	Decreased inflammation, improved neurological function, methotrexate therapy support	[141,142,143]
Vitamin C	ROS neutralization, collagen synthesis support, reduction in inflammatory markers (CRP, ESR)	Decreased cartilage degradation, reduction in CRP, improvement in connective tissue function	[146,147,148]
Vitamin K	Activation of osteocalcin and Matrix Gla-Protein (MGP), inhibition of osteoclast activity, reduction in inflammatory mediators	Improved bone mineralization, decreased inflammation, osteoporosis prevention	[149,150,151,152]

## Data Availability

Not applicable.
